# Perceived neijuan profiles and problematic short-form video use among Chinese college students: indirect associations through anxiety and escape motivation

**DOI:** 10.3389/fpsyg.2026.1963248

**Published:** 2026-10-15

**Authors:** Luyang Pi, Xinxin Mo, Shishi Li, Ranran Wang, Huanhuan Shan, Jinyun Feng

**Affiliations:** 1 Faculty of Liberal Arts and Law, Guangdong University of Petrochemical Technology, Maoming, China; 2 School of Educational Sciences, Guangdong Preschool Normal College in Maoming, Maoming, China; 3 College Student Psychological Development and Guidance Center, Guangdong University of Petrochemical Technology, Maoming, China

**Keywords:** anxiety, escape motivation, latent profile analysis, perceived neijuan, problematic short-form video use

## Abstract

Against a backdrop of intensifying competition, neijuan has become a common experience among college students and may be associated with problematic short-form video use (PSVU). However, students may experience neijuan in different ways, and the associations between these patterns and PSVU remain unclear. Drawing on the Interaction of Person-Affect-Cognition-Execution (I-PACE) model, this study identified latent profiles of perceived neijuan, compared PSVU across profiles, and examined indirect associations through anxiety and escape motivation. A convenience sample of 818 college students completed the survey. Latent profile analysis based on four dimension scores of perceived neijuan identified four profiles: low perceived neijuan (6.11%), high competition with low burden (7.70%), moderate perceived neijuan (79.71%), and high perceived neijuan (6.48%). Bolck–Croon–Hagenaars (BCH) analyses accounted for classification error in profile comparisons and in a cross-sectional serial indirect-effect model controlling for gender and grade. PSVU was lower in the low perceived neijuan and high-competition, low-burden profiles than in the moderate and high perceived neijuan profiles, whereas the latter two profiles did not differ significantly. Using the moderate profile as the reference, significant indirect associations with PSVU were observed for all three profile contrasts through anxiety, escape motivation, and the serial pathway from anxiety to escape motivation. These findings suggest that perceived competition and subjective burden may combine in different ways, with these configurations showing different associations with PSVU, anxiety, and escape motivation.

## 1 Introduction

Short-form video apps have become important channels through which college students obtain information, seek entertainment, and engage in social interaction (Liu et al., 2021; Chen et al., 2024). Features such as continuous scrolling, personalized recommendations, and immediate feedback reduce the effort required to access and switch between content. The resulting stream of novel stimuli and rewards may reinforce repeated viewing and increase the risk of problematic short-form video use (Su et al., 2021; Tian et al., 2023).

Problematic short-form video use (PSVU) refers to a difficult-to-control pattern of repeated short-form video use accompanied by adverse experiences such as withdrawal and interference with study, work, or other activities (Li et al., 2025a; Zhang et al., 2019). PSVU has been associated with a range of psychological and behavioral adjustment problems, prompting growing interest in its underlying mechanisms (Tang et al., 2026). Existing studies have primarily examined individual and family factors, including personality, self-control, family relationships, and emotion regulation (Wang et al., 2023; Zhang et al., 2024a; Liu et al., 2025a). General stress and negative life events have also been linked to PSVU (Liu et al., 2021; Liu et al., 2025b). For college students, however, stress is often embedded in persistent comparison and competition, including escalating peer effort, competition for limited opportunities and resources, and uncertainty about whether effort will yield corresponding rewards (Liu et al., 2022; Zhang et al., 2024b). General measures of stress may therefore overlook important features of these competitive and exhausting experiences.

In recent years, neijuan has become a widely used term among Chinese college students to describe excessive competition and the exhaustion associated with academic and developmental pursuits (Liu et al., 2022; Zhang et al., 2024b). Perceived neijuan comprises four dimensions—psychological pressure, social norms, competition, and resource scarcity—which capture individuals’ subjective experiences of competitive demands, group norms, and limited resources (Zhang et al., 2024b). Previous studies have associated perceived neijuan with anxiety, relative deprivation, stress responses, and psychological exhaustion (Yi et al., 2022; Liu et al., 2024; Ni et al., 2024). Research on academic neijuan has further linked such experiences with problematic smartphone use and internet gaming disorder (Gu and Mao, 2023; Wang et al., 2024). In situations characterized by persistent competition and limited resources, short-form videos may provide an accessible means of reducing negative affect or temporarily disengaging from real-life demands. Perceived neijuan may therefore represent an important psychosocial context for understanding PSVU (Gu and Mao, 2023; Sun et al., 2024).

Most previous research has treated perceived neijuan as a continuous construct and examined associations between its overall level and psychological or behavioral outcomes. However, psychological pressure, social norms, competition, and resource scarcity represent related but distinct aspects of neijuan and may combine differently across individuals (Zhang et al., 2024b). Students with similar overall levels of perceived neijuan may therefore differ in the specific configuration of these experiences. Variable-centered approaches are limited in capturing such within-person configurations, whereas person-centered approaches can identify subgroups characterized by distinct response patterns (Morin et al., 2018). Previous person-centered studies have identified heterogeneous groups in short-form video use motives, addiction-related characteristics, and self-control patterns (Gu et al., 2022; Ding et al., 2024; Liu et al., 2025a). Zhang et al. (2025) have already identified distinct neijuan configurations and their psychological correlates. The present study extends this person-centered work to PSVU and its associations with anxiety and escape motivation in a college sample.

The present study draws on the Interaction of Person-Affect-Cognition-Execution (I-PACE) model as an overarching framework for understanding the association between perceived neijuan and PSVU (Brand et al., 2016, 2019, 2025). The model highlights the roles of affective responses and application-specific motives in problematic internet-related behaviors. Accordingly, this study first used latent profile analysis to identify distinct profiles of perceived neijuan and examine differences in PSVU across profiles. It then examined anxiety and escape motivation as potential affective and motivational correlates linking perceived neijuan profiles to PSVU. By combining a person-centered perspective with an affective-motivational framework, the study aims to clarify both the heterogeneity of perceived neijuan and the affective and motivational associations with PSVU among college students.

## 2 Literature review and hypotheses

### 2.1 Theoretical framework: the I-PACE model

The Interaction of Person-Affect-Cognition-Execution (I-PACE) model provides an integrative framework for understanding the development and maintenance of specific internet-use disorders. It proposes that problematic or addictive internet use emerges from interactions among predisposing characteristics, situational triggers, affective and cognitive responses, and executive functions. Personal characteristics and situational contexts shape individuals’ emotional responses and cognitive appraisals of specific stimuli, which in turn influence use expectancies, behavioral choices, and control over particular internet applications (Brand et al., 2016, 2019).

The I-PACE model also emphasizes the gratificatory and compensatory functions of internet applications. Individuals are more likely to engage repeatedly in an online activity when they expect it to provide pleasure, alleviate negative emotions, or offer temporary relief from real-life problems. The immediate gratification obtained from use may reinforce these expectancies and behavioral tendencies, gradually making the application a habitual means of coping with particular situations or emotions. Repetition of this process may weaken control over use and increase the likelihood of problematic use (Brand et al., 2019, 2025).

Within this framework, perceived neijuan profiles represent different patterns of college students’ experiences of competition and its associated pressures. Anxiety is conceptualized as an affective response, whereas escape motivation reflects a specific motive for using short-form video apps. PSVU is treated as the behavioral outcome. Accordingly, the present study examines the associations between perceived neijuan profiles and PSVU, as well as the roles of anxiety and escape motivation in these associations.

### 2.2 Perceived neijuan profiles and problematic short-form video use

Perceived neijuan comprises four dimensions: psychological pressure, social norms, competition, and resource scarcity. These dimensions capture different aspects of college students’ experiences of excessive competition and its exhausting consequences (Zhang et al., 2024b). Psychological pressure reflects the subjective burden associated with competition; social norms refer to perceived expectations of continual effort and escalating investment; competition captures comparisons over grades, opportunities, and status; and resource scarcity reflects perceptions of limited opportunities and resources.

Although these dimensions are related, they represent distinguishable components of the neijuan experience (Zhang et al., 2024b) and may combine differently across individuals. Some students may perceive intense competition while experiencing relatively low psychological pressure and resource scarcity, whereas others may simultaneously experience strong normative demands, intense competition, resource scarcity, and psychological burden. A previous latent profile study identified distinct configurations of perceived neijuan, indicating that competition, social norms, psychological pressure, and resource scarcity do not necessarily rise or fall together (Zhang et al., 2025). Thus, students with similar overall levels of perceived neijuan may nevertheless differ in the configuration of these experiences. Latent profile analysis is well suited to capturing such heterogeneity because it identifies relatively homogeneous subgroups based on shared response patterns across multiple indicators (Morin et al., 2018).

Direct evidence comparing PSVU across perceived neijuan profiles remains limited, but related findings suggest that the two may be associated. Perceived stress has been linked to PSVU among college students, and negative life events may be associated with PSVU through emotional and cognitive processes (Liu et al., 2021; Liu et al., 2025b). Continual comparison, resource constraints, and psychological burden associated with neijuan have also been linked to stress responses, anxiety, and mental exhaustion (Yi et al., 2022; Liu et al., 2024; Ni et al., 2024). When competition is accompanied by greater psychological pressure and perceived resource scarcity, the immediate feedback, attentional diversion, and brief relaxation offered by short-form videos may become particularly appealing. Accordingly, levels of PSVU may differ across perceived neijuan profiles.

### 2.3 The indirect association through anxiety

Continual comparison, rising standards, limited opportunities, and uncertain returns on effort may heighten concerns about failure, falling behind, and future outcomes (Liu et al., 2022; Zhang et al., 2024b). College students may repeatedly monitor their peers’ effort and performance and adjust their own goals accordingly, potentially increasing feelings of uncontrollability and psychological burden (Liu et al., 2024; Zhang et al., 2024b). Perceived neijuan has been associated with anxiety, stress responses, and mental exhaustion, while resource scarcity and persistent competition may further intensify uncertainty (Yi et al., 2022; Liu et al., 2024; Ni et al., 2024). These findings suggest that different perceived neijuan profiles may be associated with different levels of anxiety.

Anxiety may, in turn, be associated with greater reliance on short-form videos. Worry, tension, and repetitive thinking can increase the tendency to seek activities that rapidly redirect attention, alleviate distress, or provide immediate pleasure (Kardefelt-Winther, 2014; Brand et al., 2019). Because short-form videos are brief, highly stimulating, and readily accessible, they can quickly capture attention and temporarily interrupt worry (Su et al., 2021; Tian et al., 2023). Previous studies have consistently linked anxiety with problematic smartphone use, problematic social media use, and PSVU (Elhai et al., 2017; Vannucci et al., 2017; Zhang et al., 2024a). Longitudinal evidence also suggests a dynamic association between anxiety symptoms and problematic short-form video use (Li et al., 2025b).

From an I-PACE perspective, affective responses to situational factors can shape appraisals of and tendencies toward specific internet applications (Brand et al., 2019, 2025). Anxiety may therefore represent an affective pathway linking perceived neijuan profiles with PSVU.

### 2.4 The indirect association through escape motivation

Escape motivation refers to the use of media to temporarily avoid real-life stress, unpleasant experiences, or unresolved problems (Baumeister, 1990; Kardefelt-Winther, 2014). Escape theory suggests that when individuals struggle with negative self-evaluations and real-life demands, narrowing attention to immediate stimuli can temporarily reduce psychological discomfort (Baumeister, 1990). Similarly, the compensatory internet use model proposes that online activities may provide short-term compensation and emotional relief when real-life needs are unmet or difficulties cannot be effectively resolved (Kardefelt-Winther, 2014).

Continual competition, limited resources, and uncertain rewards in a neijuan context may leave college students feeling exhausted or powerless (Zhang et al., 2024b; Ni et al., 2024). When such pressures cannot be quickly resolved, students may be more inclined to use media to disengage temporarily from current tasks and competitive demands (Kardefelt-Winther, 2014; Gu and Mao, 2023). Short-form video apps are particularly suited to this function because their content is brief, rapidly changing, easily accessible, and continuously recommended, allowing attention to shift quickly away from real-life concerns (Tian et al., 2023; Sun et al., 2024).

Relaxation and escape from reality are important motives for sustained online video viewing (Yang et al., 2021), and academic stress has been linked to problematic media use through escape and coping motives (Mu et al., 2022; Gu and Mao, 2023). Research on short-form videos similarly indicates a strong association between escape motivation and PSVU and suggests that escape motivation may connect stressful experiences with problematic use (Sun et al., 2024; Chen et al., 2024). Escape motivation may therefore represent a motivational pathway through which perceived neijuan profiles are associated with PSVU.

### 2.5 The hypothesized serial indirect association through anxiety and escape motivation

The I-PACE framework suggests a possible sequence through which an affective response develops into a specific motive for media use (Brand et al., 2019, 2025). Continual comparison, limited opportunities, and uncertain rewards may evoke worry, tension, and feelings of uncontrollability (Yi et al., 2022; Liu et al., 2024). As these negative experiences intensify, individuals may become increasingly motivated to seek activities that redirect attention and provide temporary relief (Kardefelt-Winther, 2014; Brand et al., 2019).

Short-form videos offer a continuous stream of brief and novel content that can facilitate temporary disengagement from worry and real-life demands (Su et al., 2021; Tian et al., 2023). From the perspectives of both the I-PACE model and the compensatory internet use model, heightened anxiety may strengthen the motivation to use short-form videos as a means of escaping real-life pressure (Kardefelt-Winther, 2014; Brand et al., 2019, 2025). Escape motivation may then increase the likelihood that short-form video use becomes a recurrent coping response and is associated with more problematic patterns of use (Sun et al., 2024).

Taken together, these perspectives support a model in which perceived neijuan profiles are associated with anxiety, which in turn is associated with escape motivation and PSVU. Given the cross-sectional design, these pathways are interpreted as indirect associations rather than evidence of temporal mediation.

### 2.6 Research hypotheses and conceptual model

Based on the preceding analysis, the conceptual model is presented in Figure 1. The following hypotheses were tested:

**Figure 1:**
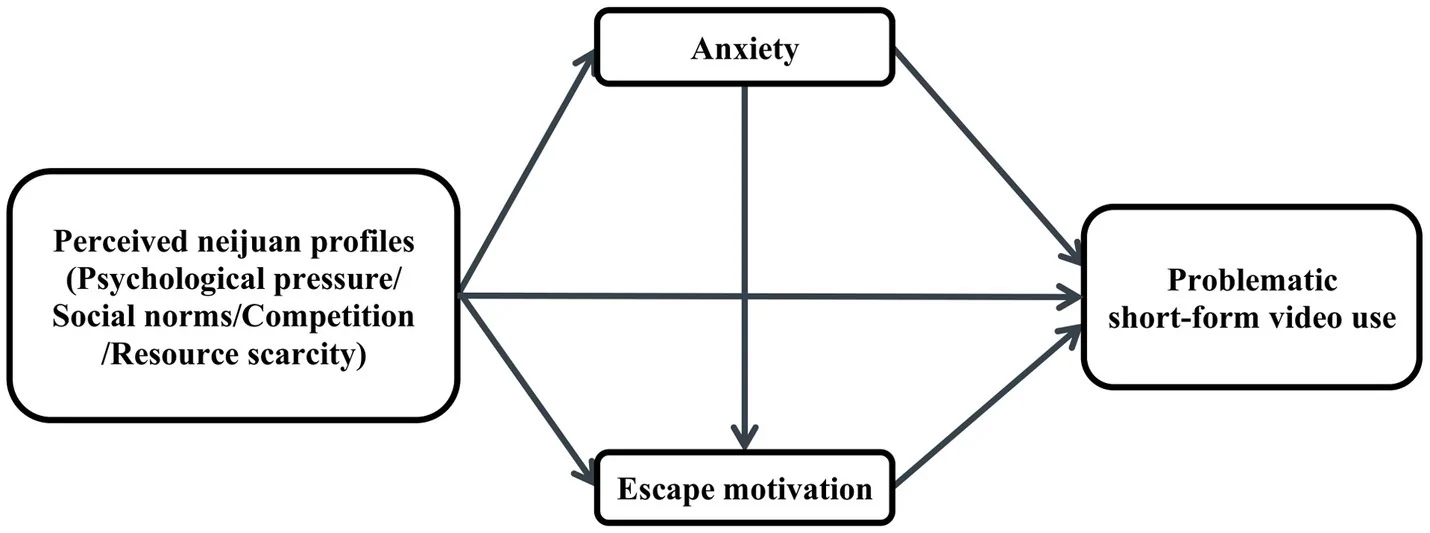
Conceptual model of the present study.

*H1*: Perceived neijuan among college students is heterogeneous. Psychological pressure, social norms, competition, and resource scarcity form several distinct latent profiles.

*H2*: College students in different perceived neijuan profiles differ significantly in their levels of problematic short-form video use.

*H3*: Anxiety has a significant relative indirect effect in the association between perceived neijuan profiles and problematic short-form video use.

*H4*: Escape motivation has a significant relative indirect effect in the association between perceived neijuan profiles and problematic short-form video use.

*H5*: Perceived neijuan profiles are associated with problematic short-form video use through a significant serial relative indirect effect involving anxiety and escape motivation.

## 3 Materials and methods

### 3.1 Participants and procedure

This cross-sectional questionnaire study recruited college students with experience using short-form video apps from two colleges in Guangdong Province, China, using convenience sampling. Participation was voluntary. Before taking part, students were informed of the study purpose, the principles of anonymity and confidentiality, and their right to withdraw at any time.

Data were collected using an anonymous online self-report questionnaire. Counselors who had received standardized training introduced the study procedures during class breaks and distributed the questionnaire link to students who agreed to participate. Participants were instructed to complete the questionnaire independently based on their actual experiences and behaviors. A total of 925 questionnaires were collected. After screening for completeness, response time, repetitive response patterns across scales, and duplicate submissions, 107 questionnaires were excluded. Response-quality screening specifically included completion in less than 3 min and selection of the same response option throughout all scales. The final analytic sample consisted of 818 participants, corresponding to a questionnaire retention rate of 88.43%. There were no missing data in the variables used for analysis.

The final sample included 259 men (31.7%) and 559 women (68.3%). By grade, 361 participants were first-year students (44.1%), 317 were second-year students (38.8%), and 140 were third-year students (17.1%). Fourth-year students were on internships and did not participate. All participants provided informed consent before completing the questionnaire. The data were anonymized and managed in accordance with confidentiality requirements.

### 3.2 Measures

#### 3.2.1 Perceived neijuan

Perceived neijuan was measured using the 18-item Perceived Neijuan Scale developed by Zhang et al. (2024b). The scale comprises four dimensions: psychological pressure, social norms, competition, and resource scarcity, with 5, 4, 5, and 4 items, respectively. Sample items for psychological pressure and social norms are “I feel frustrated in my study/work” and “Even after completing the minimum task requirements, most people around me continue to increase their effort,” respectively. Sample items for competition and resource scarcity are “People around me gain recognition from others through competition” and “Resources in my environment are so scarce that it is difficult for me to receive the rewards I deserve,” respectively. Items are rated on a 7-point Likert scale from 1 (strongly disagree) to 7 (strongly agree). The first two items are reverse scored. Higher scores indicate greater perceived neijuan. In this study, Cronbach’s *α* was 0.87 for the total scale and 0.83, 0.86, 0.93, and 0.89 for psychological pressure, social norms, competition, and resource scarcity, respectively.

#### 3.2.2 Anxiety symptoms

Anxiety symptoms were measured using the Chinese version of the 7-item Generalized Anxiety Disorder scale (GAD-7), originally developed by Spitzer et al. (2006) and validated in Chinese by He et al. (2010). The scale assesses the frequency of anxiety-related symptoms over the past 2 weeks. A sample item is “Feeling nervous, anxious, or on edge.” In the present study, responses were coded from 1 (not at all) to 4 (nearly every day), and the mean of the seven items was calculated, with higher scores indicating greater anxiety symptoms. Cronbach’s *α* was 0.92 in this study. Although the original GAD-7 uses 0–3 scoring, 1–4 coding has also been used in previous studies (Malanchini et al., 2017; Breitenstein et al., 2025). Because the present study treated anxiety symptoms as a continuous variable rather than applying clinical screening cutoffs, the 1–4 coding was retained for analysis.

#### 3.2.3 Escape motivation

Escape motivation was measured using the scale developed by Wang et al. (2020) for the context of mobile short-form video app use. This scale contains four items. A sample item is “Watching short-form videos on my phone keeps me from thinking about the problems that bother me.” Items are rated on a 5-point Likert scale from 1 (strongly disagree) to 5 (strongly agree). Mean item scores were calculated, with higher scores indicating a stronger motive to escape reality by watching short-form videos. Cronbach’s *α* was 0.91 in this study.

#### 3.2.4 Problematic short-form video use

Problematic short-form video use was measured using the 6-item Short-Form Video App Addiction Scale adapted by Zhang et al. (2019). A sample item is “I have difficulties in focusing on my study or work due to this short-form video app.” Items are rated on a 7-point Likert scale from 1 (strongly disagree) to 7 (strongly agree). The mean of the six items was used as the PSVU score. Higher scores indicate more pronounced loss of control, withdrawal experiences, and functional impairment related to short-form video use. Although the original scale uses addiction in its title, short-form video addiction has not been formally recognized as an independent diagnostic category in DSM-5 or ICD-11. Whether excessive short-form video use should be classified as a behavioral addiction also remains debated (Li et al., 2025a). Applying an addiction label directly may pathologize frequent or maladaptive use in nonclinical samples (Panova and Carbonell, 2018). The present study used a continuous self-report score rather than a clinical diagnosis. Following previous research that used the same scale (Liu et al., 2026), PSVU was therefore adopted as the less pathologizing construct label. Cronbach’s *α* was 0.89 in this study.

### 3.3 Data analysis

SPSS 26.0 was used for data management and preliminary analyses. Cronbach’s *α* was calculated to assess the internal consistency of each scale. Means and standard deviations were used to describe the main continuous variables, and Pearson correlations were calculated to examine bivariate associations. Python was used for supplementary regression analyses. Mplus 8.3 was used for latent profile analysis, confirmatory factor analysis, and classification-error-adjusted analyses. Confirmatory factor models were estimated using robust maximum likelihood (MLR), treating item responses as continuous indicators.

Latent profile analysis was conducted in Mplus 8.3 using the mean scores of psychological pressure, social norms, competition, and resource scarcity. Reverse-scored items were recoded before calculating the dimension means. Dimension scores were used to examine configurations of the four theoretical domains, consistent with the dimension-score approach used by Zhang et al. (2025). All four indicators retained the 1–7 response metric. Models with one through six profiles were estimated using robust maximum likelihood. Dimension means varied across profiles, variances were constrained equal across profiles, and within-profile covariances were fixed at zero. Models with two or more profiles used 2,000 initial random starts, 500 final-stage optimizations, and 100 initial-stage iterations. Replication of the best log-likelihood was checked.

Profile enumeration considered the Akaike information criterion (AIC; Akaike, 1987), Bayesian information criterion (BIC; Schwarz, 1978), and sample-size-adjusted BIC (aBIC; Sclove, 1987), with lower values indicating better relative fit. Significant Lo–Mendell–Rubin adjusted likelihood ratio tests (LMR-LRT) and bootstrap likelihood ratio tests (BLRT) favor a *k*-profile model over a *k* − 1 profile model (Dziak et al., 2014; Nylund et al., 2007). The BLRT used 100 bootstrap draws, with 100 initial and 20 final-stage starts for the bootstrap models. Entropy was used to describe classification separation, not to determine the number of profiles. Profile size was considered together with the distinctiveness of the additional configurations. Statistical evidence was considered alongside profile size, substantive meaning, and model parsimony (Masyn, 2013; Nylund-Gibson and Choi, 2018; Wang and Wang, 2019).

After the primary latent profile model was selected, the Bolck–Croon–Hagenaars (BCH) method was used to compare PSVU across profiles (Bolck et al., 2004). This method treats PSVU as a distal outcome and uses classification-error weights to estimate profile-specific means, thereby reducing bias associated with classification uncertainty (Asparouhov and Muthén, 2014; Bakk and Vermunt, 2016). An overall Wald *χ*^2^ test was used to determine whether mean PSVU differed across profiles. When the overall test was significant, pairwise comparisons were conducted to identify specific between-profile differences. These BCH mean comparisons were unadjusted for gender and grade.

Cross-sectional indirect associations were estimated using a BCH secondary model, retaining the classification-error weights from the selected profile model (Asparouhov and Muthén, 2021). Anxiety, escape motivation, and PSVU had profile-specific intercepts, with common regression slopes and residual variances across profiles. Anxiety was regressed on gender and grade; escape motivation on anxiety, gender, and grade; and PSVU on anxiety, escape motivation, gender, and grade. The largest profile in the selected solution served as the reference category for the profile contrasts.

Gender was coded 0 = female and 1 = male, and grade was coded 1 = first year, 2 = second year, and 3 = third year. These covariates represented demographic differences and stage of college study, consistent with evidence of gender- and academic-year-related variation in student mental health (Gao et al., 2020). For each profile contrast, the indirect association through anxiety was the anxiety-intercept difference multiplied by the anxiety–PSVU slope. The indirect association through escape motivation used the escape-motivation-intercept difference and the escape-motivation–PSVU slope. The serial association was the anxiety-intercept difference multiplied by the anxiety–escape-motivation and escape-motivation–PSVU slopes. The direct association was the PSVU-intercept difference, and the total association was the sum of the direct and three indirect components. MLR robust standard errors and delta-method 95% confidence intervals were obtained in Mplus. An interval excluding zero indicated a significant association. This ordering followed the theoretical model and did not establish temporal precedence.

A supplementary modal-assignment regression analysis followed the serial specification of PROCESS Model 6 (Hayes, 2022), with 5,000 case-resampling percentile Bootstrap intervals (Hayes and Preacher, 2014; Preacher and Hayes, 2008). Grade was additionally represented by two dummy variables in this supplementary regression analysis.

## 4 Results

### 4.1 Measurement structure and common method variance

Harman’s unrotated analysis yielded seven factors with eigenvalues greater than 1; the first accounted for 28.31% of the total variance. A confirmatory single-factor model fit poorly (*χ*^2^(560) = 11328.669, *p* < 0.001, CFI = 0.332, TLI = 0.290, RMSEA = 0.153, SRMR = 0.164). In contrast, a correlated seven-factor model representing the four perceived neijuan dimensions, anxiety, escape motivation, and PSVU showed substantially better fit (*χ*^2^(539) = 1977.779, *p* < 0.001, CFI = 0.911, TLI = 0.901, RMSEA = 0.057, SRMR = 0.061). Taken together, these results provided no indication that a single common factor adequately accounted for the observed covariation and supported the empirical distinctiveness of the measured constructs (Podsakoff et al., 2003).

### 4.2 Descriptive statistics and correlations

Means, standard deviations, and correlations for the four main variables are presented in Table 1. Perceived neijuan was positively correlated with anxiety (*r* = 0.445), escape motivation (*r* = 0.396), and PSVU (*r* = 0.359). Anxiety was positively correlated with escape motivation (*r* = 0.336) and PSVU (*r* = 0.363). The strongest correlation was between escape motivation and PSVU (*r* = 0.516). All correlations were significant at *p* < 0.001.

**Table 1:** Means, standard deviations, and correlations among the main variables (*N* = 818).

Variable	*M*	*SD*	1	2	3	4
1. Perceived neijuan	4.058	0.571	1.000			
2. Anxiety	1.630	0.550	0.445^***^	1.000		
3. Escape motivation	2.995	0.825	0.396^***^	0.336^***^	1.000	
4. Problematic short-form video use	3.531	1.114	0.359^***^	0.363^***^	0.516^***^	1.000

^***^p < 0.001.

### 4.3 Latent profiles of perceived neijuan

Table 2 presents the dimension-score models with one through six profiles. The four-profile model showed a significant improvement over the three-profile model according to the LMR-LRT (*p* = 0.011), whereas the tests comparing the five- and six-profile models with their respective k − 1 solutions were not significant (*p* = 0.506 and 0.129). Although AIC, BIC, and aBIC continued to decrease and the BLRT remained significant as additional profiles were extracted, these indices were considered together with profile size, substantive distinctiveness, and model parsimony. The four-profile solution showed good classification separation (entropy = 0.893), and its smallest profile included 50 participants (6.11%). Compared with the three-profile solution, the four-profile solution distinguished a high perceived neijuan group from the broad central group while retaining the low and high-competition, low-burden configurations.

**Table 2:** Fit indices for latent profile models using the four dimension scores.

Profiles	LogL	AIC	BIC	aBIC	Entropy	LMR *p*	BLRT *p*	Proportions
1	−4147.036	8310.071	8347.726	8322.321	—	—	—	1.000
2	−3982.353	7990.707	8051.896	8010.613	0.882	0.018	<0.001	0.117/0.883
3	−3876.796	7789.593	7874.316	7817.155	0.901	0.093	<0.001	0.084/0.061/0.855
**4**	**−3759.499**	**7564.999**	**7673.256**	**7600.217**	**0.893**	**0.011**	**<0.001**	**0.061/0.077/0.797/0.065**
5	−3700.670	7457.340	7589.132	7500.215	0.901	0.506	<0.001	0.787/0.031/0.034/0.081/0.067
6	−3658.749	7383.498	7538.824	7434.029	0.906	0.129	<0.001	0.006/0.088/0.037/0.037/0.765/0.067

The selected model is shown in bold. LogL = model log-likelihood; AIC = Akaike information criterion; BIC = Bayesian information criterion; aBIC = sample-size-adjusted Bayesian information criterion; LMR *p* = *p*-value for the Lo–Mendell–Rubin adjusted likelihood ratio test; BLRT *p* = *p*-value for the bootstrap likelihood ratio test.

Importantly, the five-profile solution did not yield a clearly new substantive configuration. Instead, it primarily subdivided the low-scoring profile into two small groups of 25 (3.06%) and 28 (3.42%) participants. These two groups differed mainly in the relative levels of competition and psychological pressure, while both remained comparatively low in social norms and resource scarcity. In contrast, the moderate, high-competition/low-burden, and high perceived neijuan configurations were largely preserved across the four- and five-profile solutions. Thus, the additional fifth profile increased model complexity while providing limited substantive differentiation.

Accordingly, the four-profile solution was retained as the most parsimonious and interpretable representation of the data, supported by the significant four-versus-three-profile LMR-LRT, adequate profile sizes, high entropy, and the absence of a substantively distinct additional configuration in the five-profile solution. The best log-likelihood was replicated for all multiprofile dimension-score models.

The low perceived neijuan profile included 50 participants (6.11%). Its conditional means were 2.795 for psychological pressure, 2.509 for social norms, 3.397 for competition, and 2.265 for resource scarcity, all of which were lower than those observed in the moderate profile.

The high-competition, low-burden profile included 63 participants (7.70%). It was characterized by relatively high levels of social norms (4.871) and competition (5.498), combined with relatively low psychological pressure (2.251) and resource scarcity (2.587). Thus, this profile reflected strong perceptions of competitive and normative demands without correspondingly high subjective pressure or perceived scarcity.

The moderate perceived neijuan profile was the largest group, comprising 652 participants (79.71%). Its conditional means were 3.812 for psychological pressure, 4.174 for social norms, 4.438 for competition, and 3.915 for resource scarcity, reflecting generally moderate levels across the four dimensions.

The high perceived neijuan profile included 53 participants (6.48%). Its conditional means were 4.377 for psychological pressure, 5.657 for social norms, 5.702 for competition, and 4.792 for resource scarcity, indicating comparatively high levels across all four dimensions.

Figure 2 shows the four dimension-score profiles. Average posterior probabilities for the assigned profiles ranged from 0.863 to 0.956 (Table 3). The four configurations supported the heterogeneity proposed in H1.

**Figure 2:**
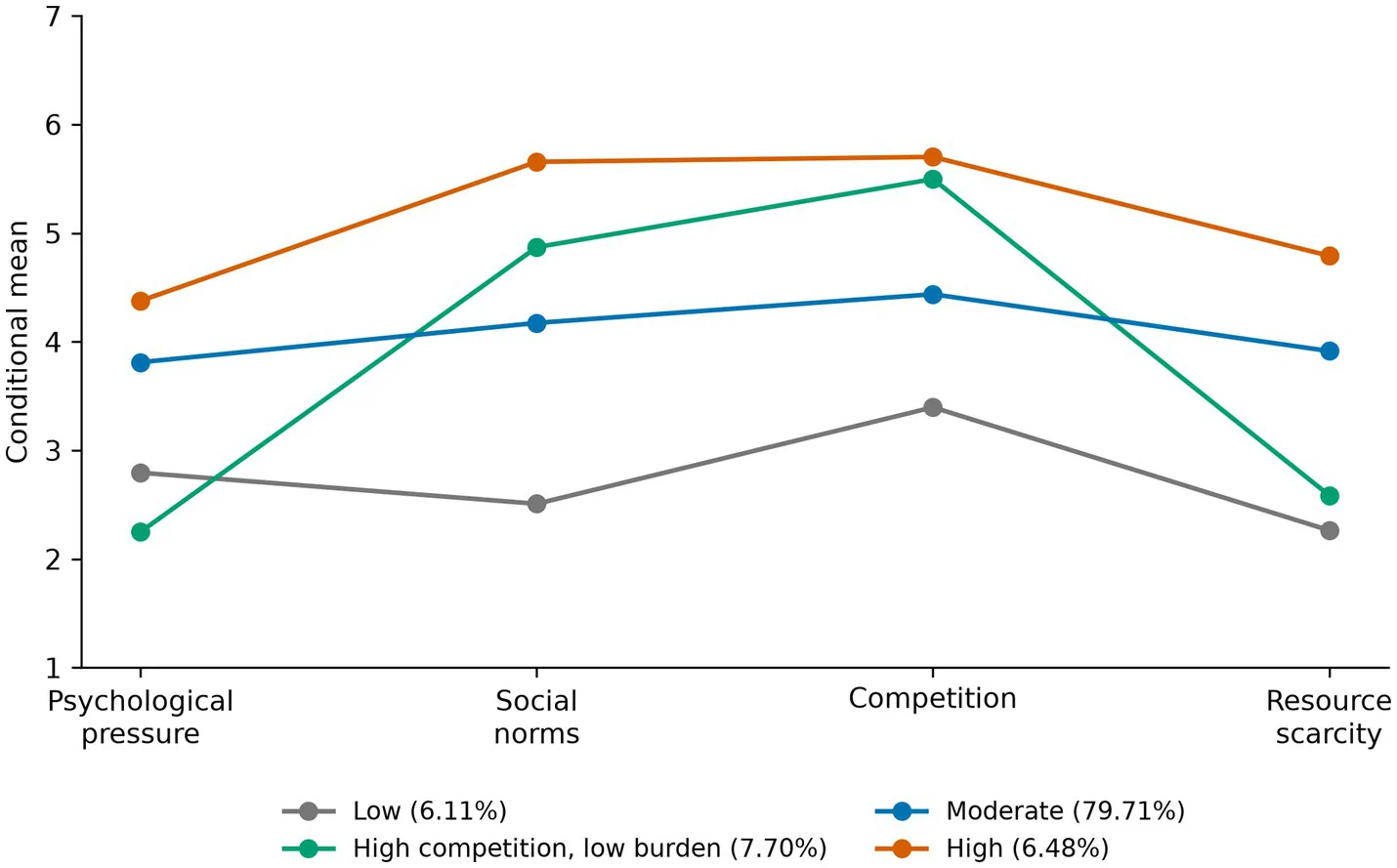
Conditional dimension means for the four-profile model of perceived neijuan (*N* = 818). Dimension means retain the 1–7 response metric. Percentages reflect most likely profile assignments.

**Table 3:** Profile sizes and average posterior classification probabilities for the four-profile model.

Profile	*n*	%	*P(C1)*	*P(C2)*	*P(C3)*	*P(C4)*
C1 Low	50	6.11	0.886	0.011	0.103	0.000
C2 High competition, low burden	63	7.70	0.004	0.863	0.124	0.008
C3 Moderate	652	79.71	0.009	0.019	0.956	0.016
C4 High	53	6.48	0.000	0.017	0.112	0.871

BCH comparisons showed an overall difference in PSVU across the four profiles, Wald *χ*^2^(3) = 132.908, *p* < 0.001 (Table 4). The means were 2.419 (*SE* = 0.172) for low perceived neijuan, 2.300 (*SE* = 0.143) for high competition with low burden, 3.710 (*SE* = 0.043) for moderate perceived neijuan, and 3.963 (*SE* = 0.191) for high perceived neijuan. Both the low and high-competition, low-burden profiles scored below the moderate and high profiles (all *p* < 0.001). The low and high-competition, low-burden profiles did not differ significantly (*p* = 0.598), nor did the moderate and high profiles (*p* = 0.206). Overall, these findings supported H2, indicating significant differences in PSVU across the perceived neijuan profiles.

**Table 4:** Differences in problematic short-form video use across perceived neijuan profiles using the BCH method.

Profile/comparison	Mean/difference	*SE*	95% CI of difference	*p*
Low	2.419	0.172	—	—
High competition, low burden	2.300	0.143	—	—
Moderate	3.710	0.043	—	—
High	3.963	0.191	—	—
Low vs. High competition, low burden	0.118	0.224	[−0.321, 0.558]	0.598
Low vs. Moderate	−1.291	0.179	[−1.643, −0.940]	< 0.001
Low vs. High	−1.544	0.256	[−2.047, −1.042]	< 0.001
High competition, low burden vs. Moderate	−1.410	0.153	[−1.709, −1.111]	< 0.001
High competition, low burden vs. High	−1.662	0.241	[−2.134, −1.191]	< 0.001
Moderate vs. High	−0.253	0.200	[−0.644, 0.139]	0.206

BCH means are unadjusted for gender and grade. Pairwise estimates represent mean differences (first profile minus second) with robust 95% confidence intervals. Pairwise *p*-values are unadjusted for multiple comparisons; all four significant contrasts remained significant after Bonferroni correction (*α* = 0.05/6).

### 4.4 Serial indirect-effect analysis

The BCH model compared the low, high-competition, low-burden, and high profiles with the moderate profile as the reference group, with gender and grade included as covariates. Table 5 and Figure 3 present the path estimates, and Table 6 presents the relative effect decomposition. All estimates in this section account for classification error in the selected four-profile solution. Covariate coefficients are provided in Supplementary Table S1.

**Table 5:** Classification-error-adjusted paths in the cross-sectional serial indirect-effect model.

Path	*B*	*SE*	95% CI	*β*	*p*
Low vs. moderate → Anxiety	−0.458	0.075	[−0.605, −0.311]	−0.833	<0.001
Low vs. moderate → Escape motivation	−0.854	0.150	[−1.148, −0.561]	−1.036	<0.001
Low vs. moderate → PSVU	−0.617	0.173	[−0.956, −0.278]	−0.553	<0.001
High competition, low burden vs. moderate → Anxiety	−0.506	0.053	[−0.610, −0.401]	−0.920	<0.001
High competition, low burden vs. moderate → Escape motivation	−0.520	0.145	[−0.804, −0.237]	−0.631	<0.001
High competition, low burden vs. moderate → PSVU	−0.885	0.143	[−1.166, −0.605]	−0.794	<0.001
High vs. moderate → Anxiety	0.550	0.117	[0.321, 0.780]	1.001	<0.001
High vs. moderate → Escape motivation	0.295	0.123	[0.053, 0.536]	0.357	0.017
High vs. moderate → PSVU	−0.178	0.203	[−0.576, 0.219]	−0.160	0.380
Anxiety → Escape motivation	0.316	0.060	[0.199, 0.433]	0.211	<0.001
Anxiety → PSVU	0.331	0.070	[0.194, 0.467]	0.163	<0.001
Escape motivation → PSVU	0.532	0.048	[0.437, 0.627]	0.394	<0.001

Estimates use BCH weights and MLR robust standard errors and 95% confidence intervals. The moderate profile is the reference. Profile-contrast *β* = *B*/SD of the dependent variable; continuous-variable *β* = *B* × SD of the predictor/SD of the dependent variable, using full-sample observed SDs. Gender and grade were controlled in all three equations. Covariate coefficients are reported in Supplementary Table S1. Gender is coded 0 = female and 1 = male; grade is coded 1–3. Covariate slopes are constrained equal across profiles.

**Figure 3:**
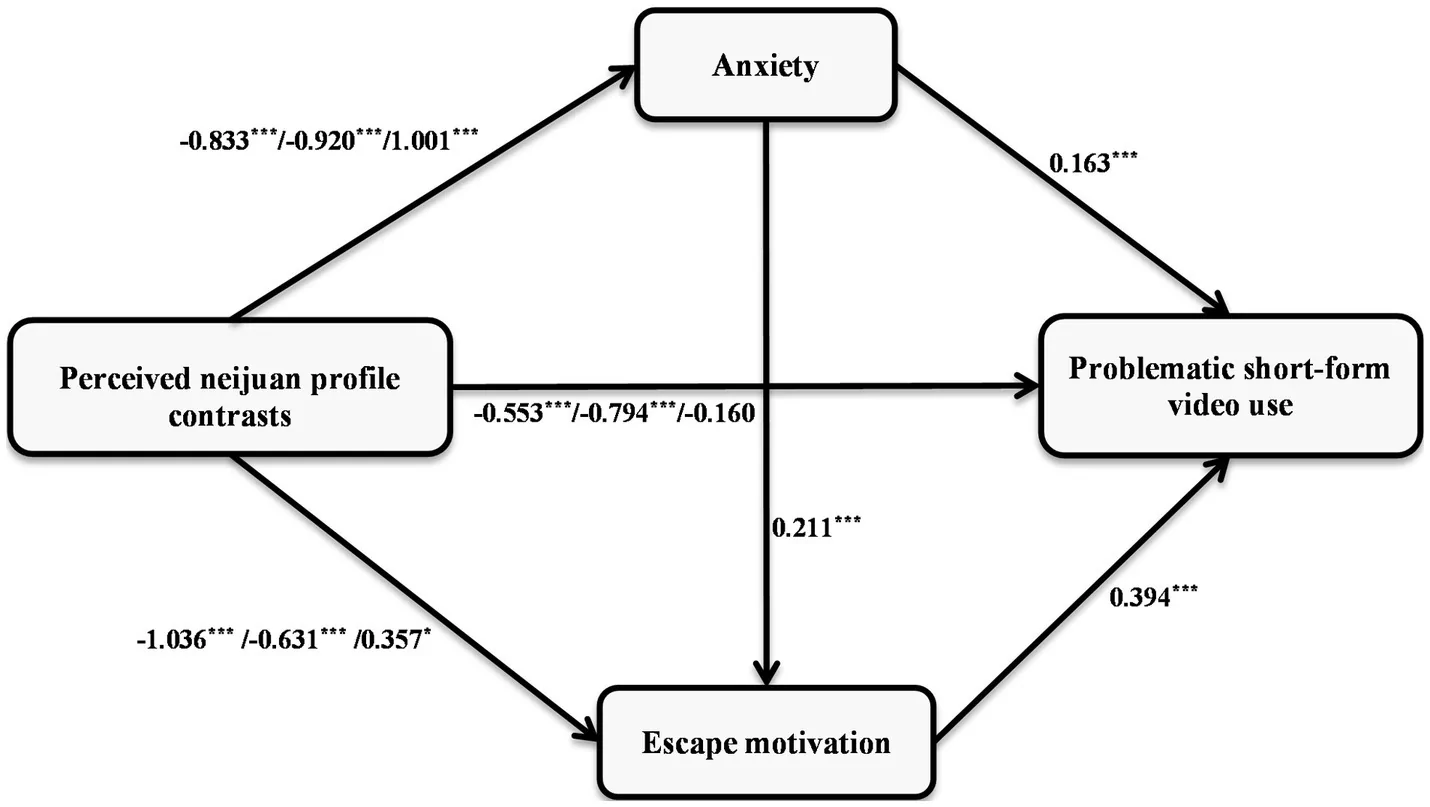
Classification-error-adjusted cross-sectional serial indirect-effect model. *β* values are standardized as defined in Table 5. For each profile path, coefficients are ordered low, high-competition/low-burden, and high, each relative to moderate. Gender and grade were included in all equations. ^*^p < 0.05, ^**^p < 0.01, ^***^p < 0.001.

**Table 6:** Decomposition of relative effects across perceived neijuan profiles.

Profile contrast	Relative association	*B*	Robust *SE*	95% CI
Low vs. moderate	Total	−1.300	0.180	[−1.653, −0.947]
Low vs. moderate	Direct	−0.617	0.173	[−0.956, −0.278]
Low vs. moderate	Via anxiety	−0.151	0.040	[−0.230, −0.073]
Low vs. moderate	Via escape motivation	−0.455	0.095	[−0.641, −0.269]
Low vs. moderate	Via anxiety → escape motivation	−0.077	0.017	[−0.109, −0.045]
High competition, low burden vs. moderate	Total	−1.414	0.154	[−1.715, −1.113]
High competition, low burden vs. moderate	Direct	−0.885	0.143	[−1.166, −0.605]
High competition, low burden vs. moderate	Via anxiety	−0.167	0.036	[−0.238, −0.097]
High competition, low burden vs. moderate	Via escape motivation	−0.277	0.080	[−0.434, −0.120]
High competition, low burden vs. moderate	Via anxiety → escape motivation	−0.085	0.019	[−0.122, −0.048]
High vs. moderate	Total	0.253	0.200	[−0.140, 0.646]
High vs. moderate	Direct	−0.178	0.203	[−0.576, 0.219]
High vs. moderate	Via anxiety	0.182	0.055	[0.074, 0.290]
High vs. moderate	Via escape motivation	0.157	0.067	[0.025, 0.288]
High vs. moderate	Via anxiety → escape motivation	0.093	0.027	[0.039, 0.146]

All estimates use BCH weights. SEs and 95% confidence intervals are based on MLR robust delta-method inference. The moderate profile is the reference. Gender and grade were controlled in all equations. Total associations are sums of the direct and three specific indirect components. Supplementary modal-assignment analyses used 5,000-resample percentile Bootstrap intervals for specific indirect associations; their results are summarized in Section 4.5 and reported in Supplementary Table S2.

Anxiety was positively associated with escape motivation (*B* = 0.316, *SE* = 0.060, *p* < 0.001). Both anxiety (*B* = 0.331, *SE* = 0.070, *p* < 0.001) and escape motivation (*B* = 0.532, *SE* = 0.048, *p* < 0.001) were positively associated with PSVU. These slopes were constrained to be equal across profiles.

Compared with the moderate profile, the low and high-competition, low-burden profiles showed lower levels of anxiety and escape motivation, whereas the high profile showed higher levels. All six contrasts were statistically significant (*p* < 0.05; Table 5). The contrasts in escape motivation were estimated conditional on anxiety and the covariates.

The low and high-competition, low-burden profiles showed significant negative relative total and direct associations with PSVU compared with the moderate profile (Table 6). For the high profile, neither the relative total association (*B* = 0.253, 95% CI [−0.140, 0.646]) nor the relative direct association (*B* = −0.178, 95% CI [−0.576, 0.219]) was statistically significant, as both confidence intervals included zero.

The relative indirect association through anxiety was significant in all three contrasts. Relative to the moderate profile, the estimates were *B* = −0.151, 95% CI [−0.230, −0.073], for the low profile; *B* = −0.167, 95% CI [−0.238, −0.097], for the high-competition, low-burden profile; and *B* = 0.182, 95% CI [0.074, 0.290], for the high profile. All three 95% confidence intervals excluded zero, supporting H3.

The relative indirect association through escape motivation was significant in all three contrasts. Relative to the moderate profile, the estimates were *B* = −0.455, 95% CI [−0.641, −0.269], for the low profile; *B* = −0.277, 95% CI [−0.434, −0.120], for the high-competition, low-burden profile; and *B* = 0.157, 95% CI [0.025, 0.288], for the high profile. All three 95% confidence intervals excluded zero, supporting H4.

The relative indirect association through anxiety and escape motivation in the specified serial order was significant in all three contrasts. Relative to the moderate profile, the estimates were *B* = −0.077, 95% CI [−0.109, −0.045], for the low profile; *B* = −0.085, 95% CI [−0.122, −0.048], for the high-competition, low-burden profile; and *B* = 0.093, 95% CI [0.039, 0.146], for the high profile. All three 95% confidence intervals excluded zero, supporting H5.

### 4.5 Supplementary analyses

In modal-assignment regressions, all nine specific indirect associations retained their directions and had 95% percentile Bootstrap intervals excluding zero (Supplementary Table S2). The same pattern remained when grade was coded using two dummy variables. In the model with grade coded 1–3, the high-versus-moderate total and direct associations were also nonsignificant. The modal-assignment and BCH analyses therefore showed a consistent pattern of indirect associations within the selected four-profile solution.

## 5 Discussion

The dimension-score analysis identified four perceived neijuan profiles: low, high competition with low burden, moderate, and high. PSVU was lower in the low and high-competition, low-burden profiles than in the moderate and high profiles. Relative to the moderate profile, all three contrasts showed separate and serial indirect associations through anxiety and escape motivation after accounting for classification error.

### 5.1 Latent profiles of perceived neijuan and differences in problematic short-form video use

The latent profile analysis identified four patterns of perceived neijuan among college students. The low, moderate, and high perceived neijuan profiles mainly differed in their overall levels of psychological pressure, social norms, competition, and resource scarcity. In contrast, the high-competition, low-burden profile combined relatively high competition and social norms with relatively low psychological pressure and resource scarcity. This finding shows that perceived neijuan involves not only differences in overall level but also different combinations of its dimensions. It is consistent with previous evidence that the components of perceived neijuan do not always vary together (Zhang et al., 2024b, 2025).

The moderate perceived neijuan profile accounted for the largest proportion of the sample, at 79.71%, and showed scores near the midpoint across the four dimensions. The low profile reported relatively low levels of psychological pressure, social norms, competition, and resource scarcity, and its PSVU was significantly lower than that of both the moderate and high profiles. The high profile showed higher scores across all four dimensions than the moderate profile. Although its mean PSVU score was numerically higher, the difference between the high and moderate profiles was not statistically significant. These findings indicate that PSVU differences across profiles were not determined by the overall level of perceived neijuan alone and may also be related to the way its dimensions were combined.

The high-competition, low-burden profile provides a particularly useful contrast. Although students in this profile reported relatively high competition and social norms, they experienced relatively low psychological pressure and resource scarcity. Their PSVU was lower than that of the moderate and high profiles and did not differ significantly from that of the low profile. The low and high-competition, low-burden profiles differed considerably in competition and social norms, but both were characterized by relatively low psychological pressure and resource scarcity. This pattern suggests that competition-related perceptions may be more closely associated with PSVU when they are accompanied by greater subjective pressure and perceived resource constraints.

Previous studies have linked academic neijuan and competitive climates with psychological pressure, relative deprivation, and mental exhaustion (Yi et al., 2022; Liu et al., 2024; Ni et al., 2024; Xu et al., 2026). When competition and normative expectations are accompanied by greater psychological pressure and resource scarcity, students may experience stronger psychological strain. In such circumstances, the immediate feedback and attentional diversion provided by short-form videos may offer a temporary way to disengage from unpleasant experiences (Su et al., 2021; Sun et al., 2024). This interpretation is also consistent with the I-PACE framework, which emphasizes the roles of affective responses and application-specific motives in problematic use (Brand et al., 2019, 2025).

The high-competition, low-burden profile also resembles the Ambitious Bee profile reported by Zhang et al. (2025), which similarly combined relatively high competition and social norms with relatively low psychological pressure and resource scarcity. In that study, this pattern was interpreted in terms of optimism about the returns to effort and the rewards of competition. Such expectations may help explain why students perceive strong competitive norms while reporting relatively little pressure or scarcity. Because the two studies used the same measure of perceived neijuan, this similarity provides further support for the possibility that competition-related perceptions and subjective burden can form distinct configurations. At the same time, Zhang et al. identified a Couch Potato profile that was not separately observed in the present four-profile solution. This difference may partly reflect differences in sample composition. Zhang et al. included participants from a broader range of ages and occupational backgrounds and examined the profile structure across Chinese and Western samples, whereas the present study focused specifically on Chinese college students. The overlap between the two studies therefore lies mainly in the separation of competition-related perceptions from subjective burden, while the specific profile structure may vary across populations.

### 5.2 The indirect association through anxiety

Anxiety showed significant relative indirect associations across all three profile contrasts. Relative to the moderate perceived neijuan profile, anxiety was lower in the low and high-competition, low-burden profiles and higher in the high perceived neijuan profile. Anxiety was also positively associated with PSVU. This pattern suggests that anxiety is closely related to how perceived neijuan is configured across profiles and to the corresponding differences in PSVU.

The low and high profiles illustrate the general association between neijuan-related burden and anxiety. Students in the low profile reported relatively low levels of psychological pressure, social norms, competition, and resource scarcity, together with lower anxiety. In contrast, students in the high profile reported elevated levels across all four dimensions and also showed higher anxiety. Competitive demands and external comparisons may increase concerns about falling behind, while perceived resource scarcity may add uncertainty about future opportunities. This pattern is consistent with previous research linking academic neijuan and competitive climates with anxiety, stress responses, and mental exhaustion among college students (Yi et al., 2022; Liu et al., 2024; Xu et al., 2026).

The high-competition, low-burden profile provides a useful contrast. Students in this profile perceived strong competition and social norms, yet reported relatively low psychological pressure and resource scarcity and also showed lower anxiety than the moderate profile. The contrast with the high profile is particularly informative because both profiles involved strong competition and social norms but differed considerably in psychological pressure, resource scarcity, and anxiety. A similar pattern was reported by Zhang et al. (2025), whose Ambitious Bee profile combined strong competition-related perceptions with relatively low psychological pressure and resource scarcity and showed more favorable psychological adjustment. Together, these findings suggest that the association between competition-related perceptions and anxiety may depend partly on whether competition is accompanied by substantial subjective burden and perceived resource constraints.

The positive association between anxiety and PSVU is also consistent with previous findings. Anxiety has been associated with short-form video addiction and problematic short-form video use in earlier studies (Zhang et al., 2024a; Li et al., 2024; Zhang et al., 2026). Within the I-PACE framework, anxiety can be understood as an affective response relevant to problematic media use (Brand et al., 2019, 2025). When worry and tension persist, short-form videos may become particularly attractive because they provide rapid attentional shifts, immediate feedback, and temporary disengagement from unpleasant emotional states. In the present study, the differences in anxiety across perceived neijuan profiles, together with the positive association between anxiety and PSVU, help explain the significant relative indirect associations through anxiety.

### 5.3 The indirect association through escape motivation

Escape motivation also showed significant relative indirect associations across all three profile contrasts. Compared with the moderate perceived neijuan profile, escape motivation was lower in the low and high-competition, low-burden profiles and higher in the high perceived neijuan profile after accounting for anxiety, gender, and grade. Stronger escape motivation was also associated with higher levels of PSVU. These findings suggest that different configurations of perceived neijuan are accompanied by different levels of escape motivation.

Students in the low profile reported lower escape motivation than those in the moderate profile. Given their relatively low psychological pressure and resource scarcity, escaping from real-life demands may have been less salient as a motive for short-form video use. This pattern is consistent with the view that escape-oriented online use becomes more relevant when individuals experience greater stress or discomfort in their offline lives (Kardefelt-Winther, 2014).

In contrast, students in the high perceived neijuan profile reported higher escape motivation than those in the moderate profile. High psychological pressure, intense competition, and pronounced resource scarcity may make temporary disengagement from real-life demands more appealing. Previous studies have identified relaxation and escape from reality as important motives for online video use and have linked stress with problematic short-form video use through escapist or compensatory motives (Yang et al., 2021; Chen et al., 2024; Sun et al., 2024). The higher escape motivation observed in the high profile is consistent with this literature.

The high-competition, low-burden profile further clarifies this pattern. Although students in this profile perceived strong competition and social norms, they reported relatively low psychological pressure and resource scarcity and also showed lower escape motivation than the moderate profile. Considered together with the high profile, this pattern suggests that competition-related perceptions may be less closely associated with the desire to escape when they are not accompanied by substantial psychological pressure and perceived resource constraints. Different configurations of perceived neijuan may therefore be associated with different motivational responses to everyday demands.

The compensatory internet use model provides a useful framework for understanding these differences. When offline experiences are stressful or emotionally taxing, online activities may provide temporary psychological compensation (Kardefelt-Winther, 2014). Short-form videos are brief, rapidly changing, and continuously recommended, making them particularly suitable for attentional diversion and short-term disengagement (Tian et al., 2023). This combination of psychological need and platform characteristics may help explain why escape motivation is more pronounced in some perceived neijuan profiles than in others.

### 5.4 The serial indirect association through anxiety and escape motivation

All three profile contrasts showed significant serial relative indirect associations through anxiety and escape motivation. Different perceived neijuan profiles were associated with different levels of anxiety, anxiety was positively associated with escape motivation, and escape motivation was further associated with PSVU. These findings suggest that anxiety and escape motivation may be jointly relevant to the association between perceived neijuan profiles and PSVU.

This pattern also helps distinguish the roles of an affective response and a use motive. Anxiety reflects worry and tension associated with competitive pressure and perceived resource constraints, whereas escape motivation reflects the tendency to use short-form videos to disengage temporarily from such experiences. According to the I-PACE model, affective responses and application-specific motives are both relevant to problematic online use (Brand et al., 2019, 2025). The present findings are consistent with this distinction, as higher anxiety was associated with stronger escape motivation, which in turn was associated with higher PSVU.

The pattern of direct and indirect associations differed across profiles. For the high versus moderate contrast, the relative indirect associations were positive, whereas the direct association was negative and nonsignificant. For the low and high-competition, low-burden profiles, negative direct associations with PSVU remained after anxiety and escape motivation were taken into account. This suggests that anxiety and escape motivation are important correlates of the profile differences in PSVU, but other psychological or behavioral factors may also be involved. Future research could examine additional processes that distinguish these perceived neijuan configurations.

### 5.5 Theoretical and practical implications

The present study extends person-centered research on perceived neijuan by linking its configurations to PSVU and to affective and motivational correlates of problematic use. The high-competition, low-burden profile is particularly informative because students in this profile reported strong competition and social norms but relatively low psychological pressure, resource scarcity, and PSVU. The low profile provides a useful comparison, as it differed substantially from the high-competition, low-burden profile in competition and social norms but showed a similarly low level of PSVU. These patterns highlight the importance of considering how different dimensions of perceived neijuan are combined rather than focusing on competition alone. The relative indirect associations through anxiety and escape motivation further connect these configurations with the affective and application-specific motivational components emphasized in the I-PACE model. These findings extend previous research on neijuan profiles by linking profile configurations to PSVU and to the affective and motivational factors associated with it.

From a practical perspective, the findings suggest that student support related to PSVU may benefit from greater attention to psychological burden and motives for short-form video use, rather than focusing only on perceived competition (Brand et al., 2019; Chen et al., 2024). Student support may be particularly relevant for those who experience substantial psychological pressure and resource constraints together with stronger anxiety or escape-oriented use motives. The high-competition, low-burden profile also indicates that strong perceptions of competition do not necessarily coincide with greater problematic use when subjective burden remains relatively low. Previous research on stress, mental exhaustion, and compensatory internet use provides a basis for examining stress management, emotion regulation, and alternative forms of leisure in future intervention studies (Liu et al., 2024; Ni et al., 2024; Kardefelt-Winther, 2014; Sun et al., 2024). Future research could further test whether targeting these factors is effective in reducing PSVU.

### 5.6 Limitations and future research

This study has several limitations. First, all variables were assessed at the same time within a cross-sectional design. The serial indirect associations therefore cannot establish temporal order or causality (Maxwell and Cole, 2007). Previous research also suggests that anxiety and problematic short-form video use may be reciprocally related (Li et al., 2025b). Future studies could use multiwave longitudinal or intensive longitudinal designs to examine the temporal relations among perceived neijuan, anxiety, escape motivation, and PSVU.

Second, the sample was recruited by convenience from two colleges in Guangdong Province. Women comprised 68.3% of the sample, and fourth-year students completing internships were not represented. The identified profiles and their proportions may therefore differ across regions, institutions, and student populations. Future studies should recruit more diverse samples to examine the stability and generalizability of the profile structure. Longitudinal data would also allow latent transition analysis to examine whether students move between profiles as their academic and employment circumstances change (Collins and Lanza, 2010; Spurk et al., 2020).

Third, the identified profiles are partly dependent on the indicators, model specifications, and sample characteristics. The selected model assumed zero within-profile covariances and equal variances across profiles, and the three smaller profiles contained only 50 to 63 participants, resulting in less precise estimates than those for the much larger moderate profile. BCH weighting accounts for classification error within the selected profile solution, while replication is still needed to establish the stability of the identified profiles. Future studies with larger and more diverse samples would provide a stronger basis for evaluating the reproducibility of the four-profile structure.

Finally, all main variables were measured using self-report instruments and may be affected by common method bias and socially desirable responding (Podsakoff et al., 2003). Future studies could combine self-report measures with platform records, screen-time data, or experience diaries to provide complementary behavioral indicators of short-form video use (Xiong et al., 2024). Additional variables proposed in the I-PACE framework, such as use expectancies and self-regulation, could also be incorporated to provide a more comprehensive account of the associations between perceived neijuan profiles and PSVU (Brand et al., 2019, 2025).

## 6 Conclusion

This study identified four patterns of perceived neijuan among college students: a low perceived neijuan profile, a high-competition, low-burden profile, a moderate perceived neijuan profile, and a high perceived neijuan profile. PSVU differed significantly across profiles, with lower levels observed in the low and high-competition, low-burden profiles than in the moderate and high profiles. Anxiety and escape motivation each showed significant relative indirect associations and also formed a significant serial relative indirect association. These findings show that different configurations of perceived neijuan are associated with differences in PSVU among college students and highlight the relevance of anxiety and escape motivation to these associations.

## Data Availability

The raw data supporting the conclusions of this article will be made available by the authors, without undue reservation.
