# Supplementary material for: Perceived neijuan profiles and problematic short-form video use among Chinese college students: indirect associations through anxiety and escape motivation
Source: Front Psychol. 2026 Oct 15;17:1963248. doi: 10.3389/fpsyg.2026.1963248 (PMC13716809; doi:10.3389/fpsyg.2026.1963248)
Supplement: Supplementary file 1 [file Table_1.DOCX]

**Online supplemental materials for**

**Perceived Neijuan Profiles and Problematic Short-Form Video Use among Chinese College Students: Indirect Associations through Anxiety and Escape Motivation**

**List of Contents of Supplemental Materials**

**Section 1** Covariate coefficients in the BCH serial indirect-effect model.	2

Table S1	2

**Section 2** Modal-assignment regression estimates under two grade codings.	3

Table S2	3

**Section 1 Covariate coefficients in the BCH serial indirect-effect model.**

**Table S1**

Covariate coefficients in the BCH serial indirect-effect model

| Outcome | Covariate | *B* | Robust *SE* | *p* |
| --- | --- | --- | --- | --- |
| Anxiety | Gender | −0.040 | 0.040 | 0.310 |
| Anxiety | Grade | −0.007 | 0.026 | 0.789 |
| Escape motivation | Gender | −0.174 | 0.057 | 0.002 |
| Escape motivation | Grade | 0.013 | 0.036 | 0.722 |
| PSVU | Gender | 0.201 | 0.070 | 0.004 |
| PSVU | Grade | −0.028 | 0.044 | 0.535 |

Note. Unstandardized coefficients are from the classification-error-adjusted manual BCH model. Robust *SE*s use MLR estimation. Gender was coded 0 = female and 1 = male; grade was coded 1 = first year, 2 = second year, and 3 = third year. Covariate slopes were constrained equal across profiles.

**Section 2 Modal-assignment regression estimates under two grade codings.**

**Table S2**

Modal-assignment regression estimates under two grade codings

| Profile contrast | Association | Grade 1–3: *B* | 95% CI | Grade dummies: *B* | 95% CI |
| --- | --- | --- | --- | --- | --- |
| Low vs. moderate | Via anxiety | −0.150 | [−0.229, −0.081] | −0.149 | [−0.229, −0.080] |
| Low vs. moderate | Via escape motivation | −0.407 | [−0.583, −0.250] | −0.404 | [−0.577, −0.243] |
| Low vs. moderate | Via anxiety → escape motivation | −0.085 | [−0.121, −0.053] | −0.084 | [−0.119, −0.052] |
| Low vs. moderate | Direct | −0.489 | [−0.761, −0.218] | — | — |
| Low vs. moderate | Total | −1.132 | [−1.432, −0.833] | — | — |
| High competition, low burden vs. moderate | Via anxiety | −0.158 | [−0.221, −0.102] | −0.156 | [−0.221, −0.101] |
| High competition, low burden vs. moderate | Via escape motivation | −0.230 | [−0.370, −0.101] | −0.226 | [−0.362, −0.099] |
| High competition, low burden vs. moderate | Via anxiety → escape motivation | −0.090 | [−0.126, −0.059] | −0.088 | [−0.124, −0.058] |
| High competition, low burden vs. moderate | Direct | −0.706 | [−0.949, −0.464] | — | — |
| High competition, low burden vs. moderate | Total | −1.184 | [−1.454, −0.914] | — | — |
| High vs. moderate | Via anxiety | 0.176 | [0.088, 0.278] | 0.172 | [0.086, 0.273] |
| High vs. moderate | Via escape motivation | 0.130 | [0.009, 0.246] | 0.119 | [0.001, 0.234] |
| High vs. moderate | Via anxiety → escape motivation | 0.100 | [0.052, 0.157] | 0.097 | [0.049, 0.153] |
| High vs. moderate | Direct | −0.177 | [−0.437, 0.083] | — | — |
| High vs. moderate | Total | 0.229 | [−0.062, 0.520] | — | — |

Note. Profiles were assigned by their highest posterior probability. The moderate perceived neijuan profile was the reference. Both models controlled for gender. The first coded grade 1–3; the second used two dummy variables for second and third year, with first year as the reference. The nine specific indirect associations in each model have 95% percentile Bootstrap confidence intervals from 5,000 case resamples. Confidence intervals for direct and total associations in the grade 1–3 model are OLS *t* intervals. Direct and total associations for the grade-dummy model are not tabulated. Modal assignment does not account for classification uncertainty; the corresponding primary BCH results appear in the main text.
